# Harnessing Nanotechnology to Enhance Essential Oil Applications

**DOI:** 10.3390/molecules30030520

**Published:** 2025-01-24

**Authors:** Hossein Omidian, Luigi X. Cubeddu, Erma J. Gill

**Affiliations:** Barry and Judy Silverman College of Pharmacy, Nova Southeastern University, Fort Lauderdale, FL 33328, USA; eg1262@mynsu.nova.edu

**Keywords:** nanoencapsulation, sustainability, controlled release, antimicrobial properties, essential oil delivery systems

## Abstract

Essential oils (EOs) are versatile natural compounds with significant antimicrobial, antioxidant, antifungal, and therapeutic properties, making them valuable in industries such as food preservation, agriculture, and healthcare. However, their inherent volatility, low water solubility, and susceptibility to environmental degradation limit their direct applications. Nanotechnology offers transformative solutions to these challenges, enhancing the stability, bioavailability, and efficacy of EOs through innovative nano systems such as nano emulsions, encapsulations, and nanocomposites. This study explores the integration of nanotechnology with essential oils, emphasizing advanced preparation techniques, physicochemical properties, and diverse applications. It highlights sustainable approaches, including eco-friendly synthesis and biodegradable carriers, which align with global trends toward green chemistry. By addressing key challenges and proposing future directions, this research underscores the potential of EO nano systems to deliver multifunctional and environmentally conscious solutions for global challenges such as food security, antimicrobial resistance, and sustainable agriculture.

## 1. Introduction

Essential oils (EOs) are natural compounds widely recognized for their diverse bioactivities, including antimicrobial, antioxidant, antifungal, and therapeutic properties. Extracted from various plant sources, EOs owe their functional versatility to bioactive components like linalool, citral, cinnamaldehyde, eugenol, and carvacrol. These attributes position EOs as valuable resources across industries such as agriculture, food preservation, pest control, and healthcare [1,2,3,4,5]. However, their direct application is hindered by limitations such as high volatility, poor water solubility, rapid degradation, and susceptibility to environmental factors like heat, light, and oxygen, which significantly reduce their shelf lives and functional efficacy [6,7,8,9,10]. Poor water solubility further hampers their bioavailability and therapeutic potential [11,12], while rapid degradation during storage exacerbates these limitations [13]. Another critical hurdle is the difficulty in achieving controlled and sustained release, which is essential for applications requiring prolonged efficacy, such as drug delivery systems [14].

Nanotechnology has emerged as a groundbreaking approach to overcoming these challenges, enabling the creation of nano systems that enhance the stability, bioavailability, and functional efficacy of EOs. Through advanced techniques such as nanoencapsulation, nano emulsions, and nanocomposites, these systems improve solubility, prolong shelf life, and enable controlled release [11,13,15,16,17]. For instance, nanoencapsulation improves stability and bioavailability in anticancer therapies [5], while nano emulsions enhance antimicrobial effectiveness and preserve food quality [18,19]. Key materials, including chitosan, pectin, and mesoporous silica nanoparticles, are commonly used in nano system fabrication, with preparation methods like ultrasonication and high-pressure homogenization enabling tailored solutions for diverse applications [20,21,22]. Moreover, nano systems contribute to sustainability efforts by providing eco-friendly solutions, such as reducing the environmental impact of pest control through targeted and efficient EO delivery [23,24].

By addressing the inherent limitations of EOs, nanotechnology enables their broader and more effective use across industries, highlighting its transformative potential in advancing the functional applications of these natural compounds.

## 2. EO Nano Systems

### 2.1. Core Components of EO Nanosystems

Prominent EOs such as cinnamon oil (rich in cinnamaldehyde), clove oil (high in eugenol), and oregano oil (containing carvacrol) are integral to nano systems, especially in applications like food preservation, pest control, and therapeutic treatments. Complementary materials enhance these core components: for instance, titanium dioxide mesoporous silica nanoparticles (MSNPs) protect EOs from oxidation while allowing for controlled release [10,17,25]. Meanwhile, zinc oxide (ZnO) and copper oxide (CuO) nanoparticles improve EO production under semi-arid conditions [26,27].

Biopolymers such as chitosan and sodium alginate are crucial stabilizing agents in nano systems. Chitosan nanoparticles significantly amplify the antimicrobial properties of EOs like garlic, cinnamon, peppermint, and clove, while sodium alginate-based nano emulsions containing oregano oil effectively suppress bacterial growth in food preservation applications [28,29,30,31]. Surfactants like Tween 80 further enhance the stability and dispersion of EO-based nano systems, exemplified by clove oil formulations [32]. Liposomal carriers made with lecithin and cholesterol are also gaining traction, as seen in garlic oil nano systems [33].

### 2.2. Applications Across Industries

**Food Preservation:** The antimicrobial activities of EOs, attributed to compounds like thymol and carvacrol (e.g., in thyme and *Zataria multiflora* oils), are effectively harnessed in nano systems to inhibit microbial growth, extend shelf life, and improve oxidative stability. These compounds disrupt microbial cell membranes, leading to leakage of ions and essential metabolites, which inhibits bacterial growth [34,35,36]. For example, thymol and carvacrol-based nano systems have been applied to yogurt drinks and labneh to reduce spoilage and maintain product quality. Similarly, eugenol from clove oil disrupts fungal cell walls and mitochondrial activity, enhancing antifungal activity in preserving pomegranate arils and refrigerated meats [29,37]. Cinnamaldehyde, known for its ability to interfere with microbial enzyme activity and cell signaling pathways, strengthens antimicrobial nanocomposite films, effectively protecting strawberries and other perishables from spoilage [15,38,39].

**Healthcare:** EOs demonstrate significant therapeutic potential when incorporated into nano systems, leveraging mechanisms such as apoptosis induction, oxidative stress modulation, and anti-inflammatory activity. For instance, beta-pinene in *Ferula gummosa* EO and cumin aldehyde in cumin EO induce apoptosis by disrupting mitochondrial membranes and activating caspase-dependent pathways, thereby exerting cytotoxic effects on colon and breast cancer cells [5,12]. The *Ferula gummosa* EO nano emulsion (FEGO-NE) inhibits angiogenesis by suppressing vascular endothelial growth factor (VEGF) signaling and enhances the expression of antioxidant genes, resulting in a 69.72% reduction in tumor volume within 14 days [5]. Additionally, eucalyptol from *Blumea balsamifera* oil reduces inflammation by inhibiting pro-inflammatory cytokine production and promotes wound healing through enhanced tissue regeneration [40]. These nano systems offer advanced therapeutic strategies for managing cancer, wounds, and oxidative stress-related conditions.

**Biofilm Inhibition and Antimicrobial Resistance:** EO nano systems effectively address biofilm formation and antimicrobial resistance through their ability to disrupt microbial adhesion, quorum sensing, and enzyme activity. For example, peppermint EO inhibits biofilm formation by interfering with bacterial signaling pathways, particularly quorum sensing, and destabilizing the extracellular polymeric substance (EPS) matrix [41]. Eugenol and cinnamaldehyde exhibit membrane-disrupting properties that impair biofilm development and promote microbial cell death [9,39]. Furthermore, EOs combat antimicrobial resistance by targeting efflux pump mechanisms and inhibiting the synthesis of essential proteins and nucleic acids. Thymol and carvacrol, delivered through nano systems, synergistically disrupt bacterial membranes, enhancing their effectiveness against resistant strains [34,35,36].

**Agriculture:** EO nano systems also contribute to sustainable agricultural practices by integrating bioactive compounds into eco-friendly pest control solutions. Citronella oil, for instance, acts as a mosquito larvicide by targeting the respiratory system of larvae, while alpha-terpinene and gamma-terpinene from *Origanum majorana* oil serve as fumigants for grain storage by disrupting insect nervous systems [42,43]. Additionally, combining EOs with nanoparticles, such as titanium dioxide with vetiver oil, enhances plant resilience under environmental stress. This approach not only mitigates stress-induced damage but also boosts essential oil production and plant health [44].

**Advanced Packaging:** EO-based nano systems can transform packaging by incorporating antimicrobial and antifungal properties that maintain food quality and safety. Compounds like cinnamaldehyde and eugenol disrupt microbial enzymes and inhibit cellular respiration, effectively reducing microbial spoilage in perishable foods such as strawberries and meat products [15,38,39]. These nano systems enable sustained release of active compounds, ensuring long-term protection and extending shelf life, while also contributing to sustainable packaging solutions.

### 2.3. Sustainability and Green Chemistry

**Figure 1:**
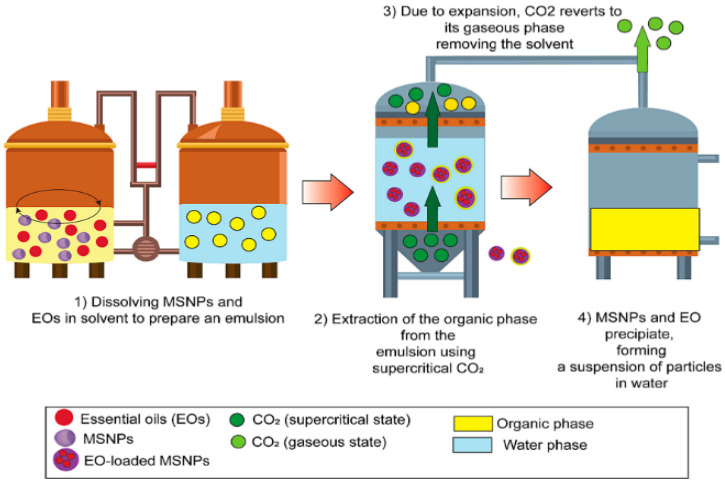
Schematic representation of the microencapsulation of EO within MSNPs using supercritical fluid extraction [10].

EO nano systems align with the principles of sustainability and green chemistry by using eco-friendly materials and processes. Techniques such as surfactant-free synthesis and biodegradable carriers minimize environmental impact while maintaining efficacy in applications like pest control and food preservation [10,29,45,46]. Supercritical carbon dioxide technology can also be used to load EOs into mesoporous silica nanoparticles (MSNPs), as shown in Figure 1. The small particle size of MSNPs enhances the surface area, allowing for efficient EO delivery, while their encapsulation efficiency ensures minimal EO loss during application, thus increasing overall efficacy. This method is particularly beneficial for ensuring stability and controlled release of EOs, making them ideal for long-term applications like food packaging and crop protection. Some of the main benefits of this technology are its simplicity, low cost, low processing time, and the use of non-reactive, nonflammable, and environmentally friendly solvents. EOs are uniformly distributed within MSNPs in a relatively short time, which enhances the encapsulation efficiency and reduces the manufacturing time [10].

Their multifunctionality allows for integrated solutions that address multiple challenges, such as combining antimicrobial properties with food safety, extending the shelf life of meat, or coupling pest management with enhanced crop productivity [28,47,48,49]. The enhanced stability of EO nano systems also ensures that their bioactive properties remain intact during storage and application, which is crucial for preserving efficacy in diverse conditions.

Table 1 outlines the chemical makeup of various essential oils (EOs) and their applications across industrial and medicinal domains. It highlights the diversity of bioactive compounds, such as citral, thymol, and eugenol, which are foundational to many EOs’ bioactivities. These compounds enable essential oils to address challenges across multiple sectors, including agriculture, food preservation, and healthcare. Thyme and oregano oils, for example, are rich in antimicrobial and anticancer properties, demonstrating their therapeutic potential [3,50]. Rosemary and orange oils extend the shelf life of food products, thus aiding in waste reduction [8,51]. Clove and cinnamon oils, with their ability to function in active food packaging and biofilm inhibition, represent cutting-edge solutions in food technology [38,50]. Ecologically, essential oils present sustainable alternatives to synthetic pesticides and preservatives. Their multifunctional nature ensures that they can meet diverse demands, from combating food spoilage to serving as natural solutions for pest control [28,42,52,53]. The table reflects a growing reliance on natural compounds for their multifunctionality, emphasizing their role in future eco-friendly and health-conscious innovations.

Table 2 categorizes the wide-ranging applications of EOs, emphasizing their role in antimicrobial activity, antifungal activity, cancer therapy, food preservation, and more. Each application is supported by references, showcasing their significance and broad utility. The prominent antimicrobial activity of EOs stands out as a core feature, enabling their use in addressing pathogens across both food and medical environments. The overlap between food preservation and antimicrobial properties illustrates their dual-purpose capability, offering industries natural and effective solutions [16,35,41,68,80]. Additionally, their antifungal properties extend this value to agriculture, where they help protect crops and stored produce from fungal infestations [84,85]. Emerging evidence supports EOs’ role in cancer therapy. Compounds in certain oils have been shown to induce apoptosis or enhance anticancer treatments, positioning EOs as promising complementary therapies in oncology [5,50,79,86]. Meanwhile, their use in mosquito and vector control highlights their environmentally friendly contributions to disease prevention [23,42]. Beyond these applications, essential oils are also noted for their stress-mitigating effects on both plants and humans. Whether supporting plant resilience against environmental stressors or promoting human relaxation, EOs exhibit dynamic and multifunctional utility [48,87,88].

## 3. Innovative Techniques for Formulating Essential Oil Nano Systems

The formulation of essential oil (EO) nano systems employs advanced techniques designed to address key challenges such as volatility, stability, and controlled release. These nano systems are tailored to enhance the bioactivity of EOs, ensuring their functionality across diverse applications. Among these approaches, nano emulsions stand out as widely used carriers, synthesized using methods such as high-pressure homogenization and ultrasonication. High-pressure homogenization produces nano emulsions with exceptionally small droplet sizes; for example, citrus EO nano emulsions with droplet sizes as small as 34 nm demonstrate prolonged stability and efficacy, making them suitable for long-term applications [61].

Ultrasonication, another prominent method, enhances the antibacterial properties of EOs like Zataria multiflora oil through precise emulsification, further validating its efficiency in improving bioactivity [35]. Solid lipid nanoparticles (SLNs) and chitosan-based carriers offer additional platforms for encapsulating EOs, ensuring improved stability and controlled release. SLNs provide excellent encapsulation and are effective in diverse applications. For example, eucalyptus oil encapsulated in SLNs exhibits enhanced antimicrobial properties, while lime oil incorporated into chitosan nanoparticles shows superior activity against foodborne pathogens [68,91]. In therapeutic contexts, cumin oil encapsulated in SLNs achieves high encapsulation efficiency, demonstrating significant cytotoxic effects in cancer therapy studies [12].

**Figure 2:**
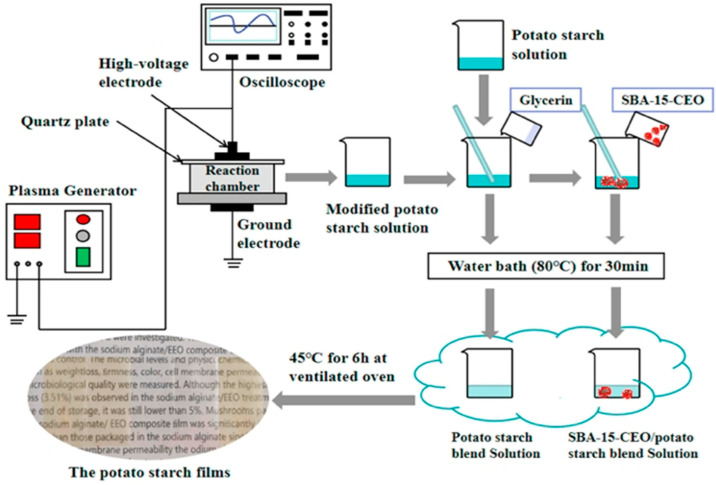
Flow chart of preparing five potato starch films. The active films based on potato starch modified by low-temperature plasma (LTP) were prepared and enriched with mesoporous nano-silica particles & cinnamon essential oil (SBA-15-CEO) [111].

Essential oils are also being integrated into functional films and coatings, expanding their utility in areas like food preservation. These films and coatings improve the stability and efficacy of EOs while offering tailored release profiles. For instance, clove oil stabilized in pullulan–gelatin films provide a controlled, slow-release profile that extends its antimicrobial efficacy [9]. Similarly, cinnamon oil incorporated into potato starch films, as shown in Figure 2, enhances thermal stability and antioxidant and antifungal activity, making it ideal for protecting perishable goods [111].

The development of electrospinning techniques has further expanded the potential applications of EOs. This method enables the encapsulation of EOs into nanofibers for various uses. Peppermint oil, for example, is encapsulated in polycaprolactone nanofibers to create antimicrobial patches designed for dermal applications [41]. In food packaging, the combination of cumin oil with zinc oxide nanoparticles improves antibacterial properties, effectively enhancing the safety and shelf life of packaged items like cheese [78].

Controlled release systems address the inherent volatility and degradability of EOs, allowing their bioactivity to be maintained over time. Mesoporous silica nanoparticles, for instance, have been used to encapsulate bergamot oil, providing sustained antidepressant efficacy through controlled release [17]. Similarly, cinnamon oil integrated into smectite clay nanoparticles offers prolonged antifungal activity against *Candida albicans*, illustrating the potential of these systems in therapeutic and agricultural settings [56].

The methodologies used to prepare EO nano systems are summarized in Table 3, which outlines the techniques, outcomes, and benefits of each approach. High-pressure homogenization and ultrasonication produce highly stable nano emulsions with uniform droplet sizes, making them particularly effective for pharmaceutical and food applications. Techniques such as ionic gelation and solvent displacement enhance encapsulation efficiency and provide precise control over release kinetics, essential for therapeutic and controlled delivery applications [29,70]. Moreover, the adoption of green synthesis methods and the use of biopolymers like pectin and chitosan highlight the growing emphasis on sustainability, aligning with global trends toward environmentally conscious technologies [47]. Advanced techniques like electrospinning and the incorporation of metallic nanoparticles add unique functionalities, including enhanced antimicrobial activity and extended shelf life [78].

## 4. Physicochemical and Functional Attributes of Essential Oil Nano Systems

The physicochemical and functional properties of essential oil (EO) nano systems are central to their effectiveness and adaptability across various applications. These attributes—such as droplet size, encapsulation efficiency, stability, and bioactivity—directly influence their performance in industries like food preservation, healthcare, and agriculture. By addressing inherent limitations of essential oils, such as volatility and degradation, nano systems enable superior functionality and prolonged efficacy.

**Droplet Size and Its Application Benefits**

Droplet size is a critical determinant of the stability and bioactivity of EO nano systems. Smaller droplet sizes enhance surface area, improving the interaction of active components with target systems, which directly contributes to their efficacy. For instance, orange peel oil nano emulsions with droplet sizes of 18.16 nm exhibit stability for up to three months under varying temperatures, demonstrating their durability and practical applicability in storage and transportation conditions [8]. Similarly, peppermint oil nano emulsions with droplet sizes as small as 9.89 nm show exceptional larvicidal activity against mosquitoes, highlighting their effectiveness in pest management [24]. These examples illustrate how optimizing droplet size directly translates to improved performance in preserving product quality, enhancing efficacy, and expanding the functional versatility of essential oils.

**Encapsulation Efficiency and Application Advantages**

Encapsulation efficiency is another pivotal property of EO nano systems, ensuring consistent delivery and controlled release of bioactive components. High encapsulation efficiency minimizes wastage, safeguards sensitive compounds from environmental degradation, and enables prolonged bioactivity, which is particularly advantageous in therapeutic and preservation contexts. For instance, Nigella sativa oil co-encapsulated with indomethacin achieves encapsulation efficiencies of up to 84%, significantly enhancing its anti-inflammatory and analgesic effects, making it valuable in medical applications [70]. Likewise, bergamot oil encapsulated in mesoporous silica nanoparticles exhibits sustained release and improved thermal stability, addressing the need for reliable and long-lasting therapeutic agents [17].

**Stability and Extended Functional Benefits**

Stability is integral to the practical use of EO nano systems, as it ensures the long-term retention of functionality during storage and application. Enhanced stability, as demonstrated by systems with high zeta potential and low polydispersity index (PDI), supports uniformity and resistance to aggregation, making nano systems suitable for industrial use. For example, thyme oil nano emulsions show inhibition zones of 10–36 mm against various pathogens, reflecting robust antimicrobial properties applicable in food preservation [34]. In agricultural applications, stability combined with innovative delivery mechanisms, as in vetiver oil with titanium dioxide nanoparticles, increases drought resistance and crop productivity, supporting sustainable farming practices [44].

**Enhanced Bioactivity and Multifaceted Applications**

The bioactivity of EO nano systems, including antimicrobial and antioxidant properties, is significantly amplified due to the nanoscale attributes. For example, Cymbopogon densiflorus oil encapsulated in nano systems shows a fourfold improvement in antioxidant IC50 values, illustrating enhanced protective benefits in applications such as food preservation and cosmetic formulations [83]. Clove oil nano systems, which extend the shelf life of chicken meatballs without altering sensory quality, highlight the role of EO nano systems in reducing food waste while maintaining product appeal [2].

Table 4 provides a detailed summary of the properties and benefits of EO nano systems, underlining their transformative impact on essential oil applications. Key attributes include particle sizes ranging from 11–264 nm, which improve solubility and absorption essential for pharmaceuticals and food products. High encapsulation efficiencies (70–96%) ensure minimal loss of active compounds and enable tailored release profiles, crucial for controlled therapeutic applications [33,70]. Enhanced thermal and oxidative stability protects essential oils during storage and processing, expanding their usability in various industries. Additionally, the amplified bioactivity of nano systems, such as stronger anti-inflammatory, antimicrobial, and antioxidant properties, directly supports their application in medical, food preservation, and agricultural sectors. Improved rheological properties further facilitate the integration of EO nano systems into diverse formulations, broadening their scope across multiple industries [104,115].

By directly linking these physicochemical properties to application-specific benefits, the utility of EO nano systems becomes clear, demonstrating their potential to address challenges and enhance performance across a spectrum of industrial applications.

## 5. Evaluation Methods for Essential Oil Nano Systems

The evaluation of essential oil (EO) nano systems relies on a comprehensive set of techniques that assess their physicochemical properties, bioactivity, and application-specific performance. These methods ensure that nano systems meet the requirements of stability, efficacy, and functionality for diverse industrial and therapeutic applications.

### 5.1. Physicochemical Characterization

Physicochemical characterization is the foundation of EO nano system evaluation, focusing on stability, morphology, and performance. Techniques such as dynamic light scattering (DLS), scanning electron microscopy (SEM), transmission electron microscopy (TEM), atomic force microscopy (AFM), and gas chromatography–mass spectrometry are commonly used to determine critical parameters like particle size, zeta potential, and polydispersity index (PDI) [32,83,121]. These metrics are essential for predicting stability and functionality in various environments. Stability tests, including encapsulation efficiency and controlled release profiles, provide further insights into the sustained performance of the nano systems [10,13]. Structural and thermal properties are evaluated using advanced techniques like X-ray diffraction (XRD), Fourier-transform infrared spectroscopy (FTIR), and thermogravimetric analysis (TGA), which assess crystallinity and thermal resilience [75,90,112,123].

### 5.2. Chemical Profiling and Molecular Interactions

**Figure 3:**
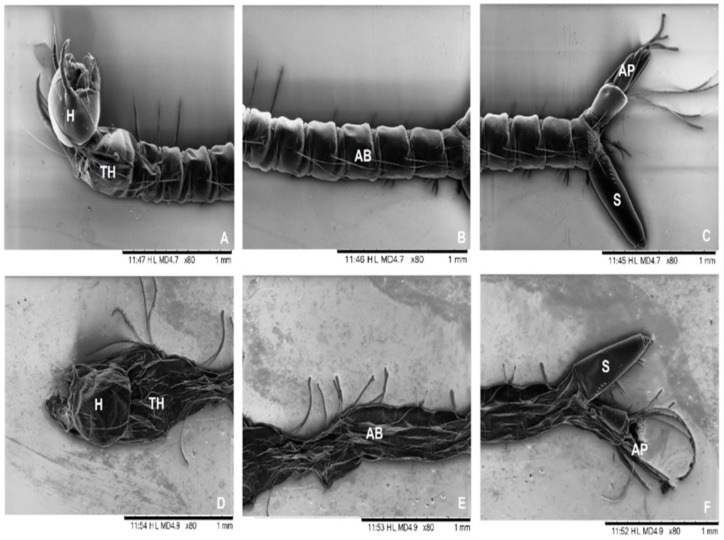
Scanning electron micrographs of Culex quinquefasciatus larvae. Normal-looking larvae in the control group (**A**–**C**). Changes shown in larvae treated with H. suaveolens nano emulsion (250 ppm) (**D**–**F**). Damage to the entire length of the cuticle, except for the siphon and the head. Head (H), thorax (TH), abdomen (AB), siphon (S), and anal papillae (AP) [104].

Understanding the chemical composition and interactions within EO nano systems is crucial for maximizing their bioactivity. Gas chromatography–mass spectrometry (GC-MS) is widely employed to profile bioactive compounds in essential oils, ensuring their integrity during nano system preparation [31,57]. Spectroscopic techniques like SEM, FTIR, UV-visible, TEM, ATR-FTIR, and Raman spectroscopy are used to identify molecular interactions and confirm the structural stability of the nano systems [73,78,79,95,104,124]. Scanning electron micrograph images shown in Figure 3 illustrates Culex quinquefasciatus larvae: normal-looking larvae in the control group (A–C); changes shown in larvae treated with H. suaveolens nano emulsion (250 ppm) (D–F); damage to the entire length of the cuticle, except for the siphon and the head; head (H), thorax (TH), abdomen (AB), siphon (S), and anal papillae (AP) [104].

### 5.3. Antimicrobial and Antifungal Activity

EO nano systems exhibit enhanced antimicrobial and antifungal activities, making them suitable for food safety, medical, and agricultural applications. Antibacterial assessments, such as minimum inhibitory concentration (MIC) and minimum bactericidal concentration (MBC) tests, determine efficacy against pathogens like *E. coli* and *S. aureus* [91,92,121]. Antifungal tests against strains like *Botrytis cinerea* and *Rhizopus stolonifera* evaluate their ability to control decay and spoilage [39,96]. Additionally, biofilm disruption studies assess the capability of nano systems to inhibit and eradicate microbial biofilms, which are critical in medical and food industries [74,125].

### 5.4. Antioxidant and Cytotoxicity Assessments

Essential oils are known for their natural antioxidant properties, often enhanced through nano system formulations. These properties are evaluated using assays like DPPH, ABTS, and CUPRAC, which quantify free radical scavenging capacity [83,90,126]. Cytotoxicity assessments are crucial for therapeutic applications, particularly in cancer research. Methods such as MTT assays, apoptosis-related gene expression studies, and Caspase-3 activity measurements are employed to determine the therapeutic potential of EO nano systems [5,101].

### 5.5. Food Preservation and Sensory Quality

EO nano systems contribute significantly to extending the freshness and safety of perishable food items. Microbial quality and spoilage reduction are evaluated through high-throughput sequencing and monitoring spoilage organisms during storage [32,49]. Sensory and biochemical assessments, including measurements of firmness, pH, and titratable acidity, provide insights into the preservation of food quality [59,127]. These evaluations ensure that EO nano systems maintain both the safety and sensory appeal of food products.

### 5.6. Agricultural Applications

In agriculture, EO nano systems enhance plant resilience and provide eco-friendly pest management solutions. Plant health under stress conditions, such as drought or salinity, is assessed through metrics like growth performance, photosynthetic efficiency, and enzymatic activity [44,122]. For pest control, larvicidal, pupicidal, and ovicidal activity tests, along with acetylcholinesterase (AChE) inhibition assays, measure the effectiveness of EO nano systems against pests [100,104].

### 5.7. Therapeutic Applications

EO nano systems are also evaluated for drug delivery, wound healing, and anti-inflammatory treatments. Wound healing assessments include histological analyses, gene expression studies, and measurements of wound closure rates [40,120]. For cancer therapy, apoptosis detection methods such as flow cytometry, AO/PI staining, and in vivo tumor inhibition studies are conducted to evaluate their potential as complementary or primary therapeutic agents [5,12].

### 5.8. Packaging and Material Applications

The functional properties of EO nano systems make them ideal for food and therapeutic packaging. Mechanical properties, such as tensile strength and elasticity, are tested to ensure robustness, while barrier properties, including gas permeability and hydrophobicity, are assessed for their effectiveness in preserving packaged goods [9,102,111]. These properties contribute to maintaining product integrity and extending shelf life.

### 5.9. Stability and Optimization Studies

Ensuring long-term durability and performance is essential for EO nano systems. Stability tests, such as thermodynamic assessments and release kinetics evaluations, are performed using techniques like high-pressure homogenization and ultrasonic emulsification [8,61]. Formulation optimization is achieved through statistical approaches like response surface methodology (RSM) and Box–Behnken Design (BBD), enabling precise refinement of nano system properties [13,36].

### 5.10. Advanced Analytical and Environmental Assessments

Cutting-edge analytical techniques provide deeper insights into EO nano system behavior. Molecular docking studies and enzymatic assays elucidate bioactivity interactions at the molecular level [92]. Cellular effects are explored using gene expression analyses, particularly in therapeutic contexts [62]. Beyond traditional applications, EO nano systems are evaluated for their environmental safety and material durability. For example, corrosion-resistance tests using electrochemical impedance spectroscopy assess their potential for protecting materials like titanium dental implants [123]. Sustainability and environmental impact studies further ensure the safety of these nano systems for broader applications [10,13].

The evaluation methods for EO nano systems provide a comprehensive framework for understanding their physicochemical, functional, and application-specific properties. These techniques ensure that EO nano systems meet the demands of stability, efficacy, and safety across industries, from food preservation to therapeutic and agricultural applications. By integrating advanced analytical approaches and sustainability considerations, EO nano systems continue to expand their potential as innovative solutions for modern challenges.

## 6. Breakthroughs in Essential Oil Nano System Applications

**Figure 4:**
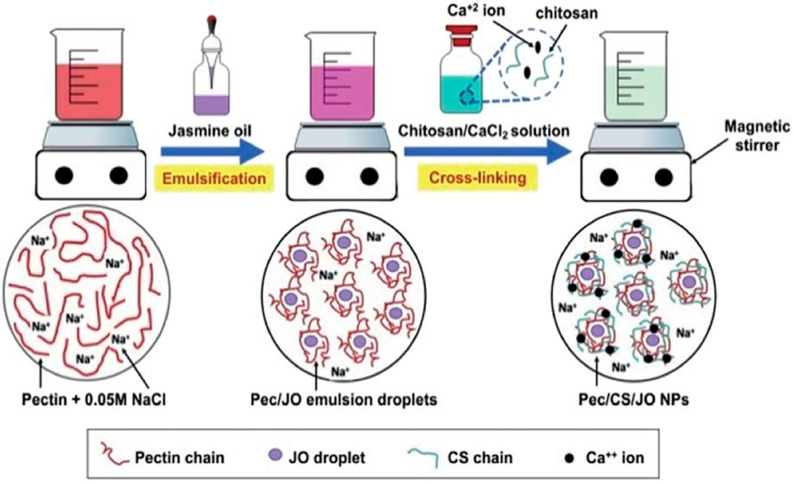
Schematic presentation of JO nano-encapsulation. The nano encapsulated JO (jasmine oil), chitosan & pectin (Pec/CS/JO NPs) were formulated using Box–Behnken Design (BBD) to ensure an optimum nanoformulation is produced. Pec/CS/JO NPs were prepared via emulsification followed by ionic gelation method [11].

The integration of nanotechnology with essential oils (EOs) has transformed their functionality and broadened their applications across multiple industries. By addressing the inherent challenges of volatility, poor stability, and rapid degradation, nanoencapsulation has enhanced the stability, bioavailability, and efficacy of EOs. For instance, jasmine EO encapsulated in chitosan/pectin nanoparticles exhibited remarkable thermal stability, as shown in Figure 4 [11], while garlic EO encapsulated in liposomal nanocarriers demonstrated improved bioactive compound preservation [33].

Advanced nano systems, such as chitosan-selenium hybrids [87] and solid lipid nanoparticles [68], enable controlled release and long-term functionality under commercial storage conditions.

EO nano systems have proven multifunctional, finding applications in agriculture, food preservation, pest control, and medicine. Their therapeutic potential is exemplified by the synergistic effect of *Teucrium polium* EO with oxaliplatin, which enhanced colon cancer treatment [86], and jasmine EO nanoformulation, which showed significant anticancer activity against MCF-7 breast cancer cells [11]. In agriculture, selenium and silicon dioxide nanoparticles mitigate heavy metal stress and increase EO yields, demonstrating their utility in sustainable farming practices [72,87]. Furthermore, EO-based nano systems provide eco-friendly antimicrobial and antifungal solutions, reducing dependence on synthetic chemicals [23,80].

Nano formulations enhance targeting and efficacy, enabling precise applications. Bergamot EO-loaded nanocarriers exhibit superior anti-inflammatory properties, making them effective for treating skin conditions [115]. Agricultural nano emulsions mitigate stress factors like salinity and drought, improving crop health and yield [4,48]. These advancements highlight the adaptability of EO nano systems to diverse challenges. Sustainability and green chemistry are integral to the development of EO nano systems. For example, Russian knapweed EO facilitated the surfactant-free synthesis of iron oxide nanoparticles [45], while bioactive glass nanoparticles provided eco-friendly solutions for agriculture and healthcare applications [80]. Such approaches not only promote environmental safety but also reduce reliance on synthetic chemicals.

In food preservation, EO nano systems have significantly extended shelf life while maintaining product quality. EO-enriched coatings have reduced microbial spoilage in meat [37,49], fish [63], and fresh produce such as table grapefruit [85] and tomatoes [7]. Cinnamon EO nano emulsions effectively prevented fungal decay in strawberries [39], and oregano EO preserved the sensory and microbial quality of sausages [47]. These applications illustrate the value of nano systems in improving food safety and extending product freshness.

Nano systems also offer dual functionality in biomedical applications, combining antimicrobial and anticancer properties. Clove and thyme EO nano emulsions showed enhanced cytotoxicity against breast cancer cells compared to taxol [50], while *Ferula gummosa* EO nano emulsions significantly reduced tumor volume in colon cancer models [5]. Aloe vera-coated eucalyptus EO nanoparticles accelerated wound healing by enhancing collagen deposition and reducing inflammation [69].

Advanced delivery systems have further optimized the sustained release and efficacy of EOs. Mesoporous silica nanoparticles enabled controlled release of cinnamon EO while maintaining its antifungal activity [111]. Pullulan-based coatings offered antioxidant protection for perishable goods such as pork loin [9,22]. These technologies maximize the functional potential of EOs in diverse applications.

EO nano systems have transformed pest control by providing sustainable alternatives to synthetic pesticides. Citronella EO nano emulsions effectively controlled beetle pests [53] and mosquitoes [100], while EO-loaded nanoparticles protected stored grains from fungal contamination and aflatoxin production [55,96]. Nanoparticles such as TiO₂ and SiO₂ enhance crop resilience and EO yields, further supporting sustainable agriculture [48,109].

Enhanced antimicrobial and antioxidant activities are consistently achieved with EO nano emulsions. Citrus EO nano emulsions exhibited an 86-fold increase in antimicrobial efficacy against *Bacillus subtilis* [61], while *Cymbopogon* EO demonstrated improved antioxidant potential [83]. These breakthroughs position EO nano systems as effective, natural alternatives for applications in food safety, healthcare, and cosmetics.

Incorporating EOs into nanocomposites has improved the mechanical, thermal, and antimicrobial properties of materials. For instance, kesum EO in gelatin–chitosan films enhanced food preservation [88], while starch films containing cinnamon EO provided superior gas barrier properties [111]. These innovations expand the scope of EO nano systems in packaging, agriculture, and biomedicine.

EO nano emulsions also offer sustainable solutions for vector control and disease prevention. Peppermint EO nano emulsions have shown promise in malaria vector management [24], and *Ferula gummosa* EO has proven effective against fungal pathogens and cancer cells [5,73]. These applications reduce environmental, and health risks associated with traditional methods.

Safety and consumer acceptability are integral to these advancements. EO nano formulations maintain the sensory qualities of food products [36,63] and exhibit negligible toxicity, ensuring safety for human and environmental health. Their applications in food preservation, healthcare, and agriculture underscore their potential as sustainable, consumer-friendly solutions.

Table 5 outlines the benefits of taste-masking technologies, focusing on their ability to suppress undesirable flavors, protect active compounds, and improve the sensory experience for diverse consumer groups. The table highlights how taste-masking technologies improve product appeal and widen the market for functional foods, beverages, and pharmaceuticals. By suppressing bitterness and preserving delicate aromas, these technologies ensure a more pleasant consumption experience, particularly for bioactive compounds with inherently strong flavors. Layered encapsulation methods not only mask tastes but also prevent flavor degradation over time, enhancing product stability and shelf life. The suitability for sensitive consumers, such as children and older adults, makes these products more inclusive and accessible [7,8,91]. Additionally, the dual functionality of combining taste masking with food stabilization demonstrates their multifaceted utility. The potential to adapt these systems across various formats—beverages, coatings, and solid foods—further broadens their commercial applications [3,47]. This innovation drives the integration of functional bioactive into mainstream products, offering healthier, more palatable options without compromising sensory quality or therapeutic efficacy.

## 7. Limitations and Future Directions for Essential Oil Nano Systems

### 7.1. Formulation, Scalability, and Integration Challenges

Developing essential oil (EO) nano systems faces several technical, economic, and scalability barriers. Maintaining consistent particle sizes, droplet stability, and batch reproducibility is particularly challenging. For instance, nano systems formulated with *Carum carvi* and *Carlina acaulis* oils often exhibit variability in their physicochemical properties [42,93]. High production costs, driven by expensive materials like mesoporous silica nanoparticles, bovine serum albumin (BSA)-dextran sulfate (DS) conjugate, and advanced equipment such as high-pressure homogenization, electrospinning, and ultrasonication systems, further hinder their broader adoption [41,46,111,120]. Incorporating biodegradable carriers like chitosan or zein into scalable systems remains difficult, as these materials often require careful handling and alignment with industrial processes [14,47,49]. Moreover, the stability of EO nano systems under variable environmental conditions continues to be a limiting factor in their long-term application [58,117].

To overcome these challenges, fostering interdisciplinary collaboration between material scientists, process engineers, and industrial stakeholders is essential. Collaborative efforts can streamline the integration of EO nano systems into production pipelines by bridging knowledge gaps and aligning formulation innovations with industrial requirements.

### 7.2. Toxicity, Environmental, and Regulatory Concerns

Despite their promising bioactivity, EO nano systems face scrutiny over their long-term safety and environmental impact. Concerns about nanoparticle bioaccumulation and ecosystem interactions, particularly with titanium dioxide, selenium, and silver nanoparticles, underscore the need for thorough ecological assessments [87,107,118]. Limited studies on biodegradability and allergenicity, combined with a lack of harmonized regulatory frameworks, contribute to consumer skepticism [21,62,108,110]. Regulatory agencies require more robust safety protocols and labeling standards to mitigate uncertainties around nanoparticle accumulation in consumables [64,92,110]. Addressing these challenges will require comprehensive safety studies and the establishment of globally accepted regulatory guidelines [13,55].

Future research initiatives should integrate cross-disciplinary expertise in toxicology, environmental sciences, and regulatory affairs to develop standardized protocols that ensure both safety and public trust. Enhanced industrial collaboration is critical to aligning safety assessments with commercialization goals, promoting faster regulatory approvals.

### 7.3. Limited Field Validation and Mechanistic Insights

While laboratory research on EO nano systems has yielded significant advancements, real-world applications remain underexplored. Field trials in clinical and agricultural settings are scarce, limiting the validation of nano systems’ efficacy in practical scenarios such as pest control, medical treatments, and food preservation [28,46,115]. Additionally, a deeper understanding of the molecular mechanisms behind the antimicrobial, antifungal, and anticancer properties of EO nano systems is needed to refine their formulations for specific applications [33,75].

Interdisciplinary research combining molecular biology, materials science, and field practitioners is necessary to close these gaps. Partnering with industry can further enhance field validation efforts, providing scalable solutions tailored to specific industrial challenges.

### 7.4. Scaling Production and Cost Efficiency

Industrial-scale production of EO nano systems encounters obstacles related to cost efficiency, batch consistency, and reproducibility. Techniques such as ultrasound emulsification, high-pressure homogenization method, and micro fluidization are energy-intensive and expensive, making them inaccessible in low-resource settings [27,61,63]. Simplifying production methods while maintaining efficacy is crucial for scaling up these technologies. Developing cost-effective alternatives will be particularly important for emerging markets, where economic barriers limit adoption [32,37,92].

Integrating industrial feedback during the development phase can ensure that production technologies align with operational realities, fostering cost-effective scale-up. Collaborative innovation between academia and industry is pivotal to addressing cost and accessibility concerns, especially in resource-limited regions.

### 7.5. Opportunities for Advancement

The future of EO nano systems lies in eco-friendly synthesis and the integration of sustainable materials. Biodegradable carriers like chitosan, pullulan, pectin, poly (ε-caprolactone), and iron oxide nanoparticles align with sustainability goals and reduce environmental risks [11,45,70]. Green synthesis techniques, surfactant-free formulations, and renewable nanomaterials offer pathways to reduce production costs and mitigate environmental concerns [43,78,93]. Expanding clinical and field validation will bridge the gap between laboratory successes and practical applications in healthcare, agriculture, and food systems [42,51,70,72].

Tailoring EO nano systems for specific applications, such as targeting bacterial strains or cancer cells, will maximize their therapeutic and functional potential. Advances in slow-release formulations and predictive modeling technologies, like AI-driven optimization, can refine design strategies and improve performance [20,98,120]. Multifunctional nano systems integrating pest control, crop enhancement, and antimicrobial properties present significant opportunities to address global challenges in food security and healthcare [27,50,116].

Interdisciplinary research alliances can expedite these advancements by merging expertise in nanotechnology, artificial intelligence, and industrial applications. Partnerships with industry stakeholders will ensure that EO nano systems are designed with end-user needs in mind, enabling seamless integration into global markets.

### 7.6. Towards Sustainable and Integrated Applications

EO nano systems are poised to play a transformative role across industries, addressing critical challenges in health, agriculture, and food security. Dual-functional solutions—such as combining pest control with crop enhancement—exemplify their potential for integrated outcomes [4,10,49].

Continued interdisciplinary collaboration, regulatory clarity, and investments in scalable, eco-friendly production methods will be pivotal in unlocking their full potential. Joint ventures between academia and industry can drive innovation while ensuring that sustainability and scalability are prioritized in future EO nano system designs [62,75,124].

## 8. Conclusions

The integration of nanotechnology with essential oils (EOs) represents a transformative advancement, overcoming the limitations of EOs while expanding their applications across critical industries. By leveraging techniques like nanoencapsulation and ultracentrifugation, EO nano systems achieve enhanced stability, controlled release, and improved bioavailability. Nanoencapsulation not only protects sensitive EO components from environmental degradation but also enables their targeted and sustained release, enhancing their therapeutic efficacy. Similarly, ultracentrifugation facilitates the precise separation of nanoscale components, ensuring uniform particle size distribution and optimized bioactivity. These advancements make EO nano systems highly effective for applications in food preservation, agriculture, and therapeutics.

Despite significant progress, challenges such as scalability, cost efficiency, regulatory compliance, and long-term safety must be addressed to ensure widespread adoption. The future of EO nano systems lies in innovative approaches, such as AI-driven optimization of nano system design and green synthesis techniques, to create multifunctional solutions tailored to global needs. By aligning these advancements with sustainability goals, EO nano systems hold the potential to redefine natural product applications, offering pathways to healthier, safer, and more sustainable industries.

## Figures and Tables

**Table 1:** Key components of essential oils across key sectors.

Essential Oil	Key Chemical(s)	Application/Focus	References
Lavandula angustifolia	Linalool	Antibacterial, thermal stability enhancement, eco-friendly pesticide formulations	[1,54]
Cymbopogon citratus (Lemongrass)	Citral	Antifungal, antioxidant, mitigating salt stress, enhancing shelf life of foods	[3,4,55]
Cinnamomum zeylanicum (Cinnamon)	Cinnamaldehyde	Antimicrobial, antifungal, insecticidal, food preservation, packaging material	[15,38,39,52,56]
Mentha spicata (Spearmint)	Menthol	Antimicrobial, therapeutic, insecticidal, growth-promoting effects, eco-friendly vector control	[57]
Eugenia caryophyllata (Clove)	Eugenol	Antioxidant, antimicrobial, antifungal, food preservation, active food packaging	[2,9,29,32,58,59]
Origanum vulgare (Oregano)	Carvacrol	Antimicrobial, anticancer, food preservation, pest control	[22,31,47]
Thymus vulgaris (Thyme)	Thymol	Antimicrobial, antifungal, food preservation, packaging, anticancer activity	[34,35,60]
Citrus sinensis (Orange)	D-Limonene	Antimicrobial, antioxidant, food preservation, shelf-life extension	[7,8,61]
Zataria multiflora	Thymol, Carvacrol	Antibacterial, anticancer, biofilm inhibition, food preservation	[35,62,63,64]
Rosmarinus officinalis (Rosemary)	1,8-Cineole, Rosmarinic acid	Antioxidant, antibacterial, anti-inflammatory, shelf-life extension	[51,65]
Cymbopogon martinii (Palmarosa)	Citral	Antimicrobial, antioxidant, shelf-life extension	[66]
Protium heptaphyllum	p-Cymene, Alpha-pinene	Larvicidal activity against Aedes aegypti	[67]
Eucalyptus globulus	1,8-Cineole, Alpha-pinene	Antimicrobial, antifungal, wound healing	[68,69]
Nigella sativa	Thymoquinone	Anti-inflammatory, anticancer, synergistic nanoformulation applications	[70]
Myristica fragrans (Nutmeg)	Myristicin	Antimicrobial, aflatoxin inhibition	[71]
Mentha arvensis (Field Mint)	Menthol	Growth enhancement, stress mitigation, improved essential oil yield	[25,72]
Ferula gummosa	Beta-pinene	Anticancer, antifungal, shelf-life extension	[5,73]
Cymbopogon khasianus	Geraniol	Antifungal, aflatoxin mitigation, food preservation	[21,55]
Satureja khuzistanica	Carvacrol, Thymol	Antibacterial, anticancer, antifungal, biofilm inhibition	[74,75]
Cuminum cyminum (Cumin)	Cumin aldehyde	Antibacterial, growth promotion, anticancer, cheese storage, active packaging	[12,76,77,78]
Anethum graveolens (Dill)	Carvone	Flower vase life extension	[77]
Lippia citriodora (Lemon Verbena)	Citral	Apoptosis induction in melanoma cells	[79]
Melaleuca armillaris	1,8-Cineole	Antibacterial, antifungal	[80]
Trachyspermum ammi (Ajowan)	Thymol	Larvicidal, acetylcholinesterase inhibition, shelf-life enhancement	[81,82]
Cymbopogon densiflorus	Trans-p-Menta-dienols	Antioxidant activity	[83]
Vetiveria zizanioides	Khusimol	Photosynthesis enhancement, essential oil production	[44]

**Table 2:** Essential oil applications.

Application	Description	References
Antimicrobial Activity	Targeting pathogens, foodborne microorganisms, or biofilm-forming bacteria in medical and food settings	[2,7,9,15,16,19,31,33,41,50,51,57,61,69,71,80,89,90,91,92]
Antifungal Activity	Inhibiting fungal growth in food preservation, agriculture, and storage environments	[2,14,21,29,39,41,55,58,59,60,75,84,85,93,94,95,96]
Insecticidal Activity	Controlling agricultural pests or disease vectors, including eco-friendly mosquito control	[1,28,30,42,43,46,52,53,77,81,82,97,98,99,100]
Cancer Therapy	Enhancing anticancer efficacy, inducing apoptosis, and improving therapeutic outcomes	[5,11,12,62,73,79,86,101]
Food Preservation	Extending shelf life, reducing microbial contamination, or maintaining sensory qualities	[7,8,15,22,37,38,47,49,58,59,63,89,93,102]
Mosquito and Vector Control	Larvicidal, pupicidal, ovicidal, and repellent activities against mosquitoes and other vectors	[23,24,53,67,99,100,103,104]
Shelf-Life Extension in Agriculture	Improving freshness and quality of fruits, vegetables, and agricultural products	[14,59,60,63,66,84,85,93]
Antioxidant Activity	Neutralizing oxidative stress, enhancing stability of oils, or improving product shelf life	[19,44,50,57,65,83,90,105]
Stress Mitigation in Plants	Alleviating stress from heavy metals, salinity, or drought, while enhancing essential oil yield	[25,26,48,72,87,106,107,108,109]
Wound Healing	Promoting tissue repair, antibacterial activity, and reducing inflammation	[40,69,110]
Packaging and Coating Applications	Development of active, edible, or antimicrobial films to protect and preserve food	[3,15,38,49,66,78,88,111,112,113]
Pest Management in Agriculture	Natural and eco-friendly alternatives for pest control	[1,28,42,43,46,52,53,81,97]
Biofilm Inhibition	Disrupting biofilm formation of pathogens in medical or food settings	[49,50,56,74,114]
Therapeutic Applications	Antidepressant, antibacterial, anti-inflammatory, or analgesic effects	[17,65,70,80,115]

**Table 3:** Preparation and processing techniques for essential oil nano systems.

Preparation/Processing Technique	Description	References
High-Pressure Homogenization	Produced nano emulsions with particle sizes <100 nm; effective for stable, transparent emulsions.	[20,37,42,61,97]
Ultrasonication/Ultrasound nano emulsification	Reduced droplet size and enhanced stability; widely used for food, cosmetics, and pharmaceuticals.	[8,13,15,21,22,24,35,57,82,95,116,117]
Micro fluidization	Created nano emulsions with narrow size distributions and enhanced bioactivity.	[6,32]
Ionic Gelation	Common for chitosan nanoparticles; provided controlled release and improved encapsulation.	[11,16,73,114]
Solvent Displacement	Optimized for small droplet sizes and uniformity; applied in food preservation and medical use.	[105]
Emulsion–Solvent Evaporation	Achieved high encapsulation efficiencies for EOs and co-delivery with drugs.	[33,70]
Response Surface Methodology (RSM)	Applied to optimize emulsification parameters like droplet size, stability, and viscosity.	[8,11,105]
Phase Inversion	Used for thermodynamically stable emulsions with small, uniform droplets.	[83]
Foam-Mat Freeze-Drying	Preserved nano emulsion properties over extended storage times; enhanced thermal stability.	[13]
Hydrophilic–Lipophilic Balance Optimization	Balanced emulsifiers to enhance stability and reduce droplet size; effective for antibacterial systems.	[57,117]
Combination with Nanoparticles	Silver, selenium, zinc, copper, or titanium oxide nanoparticles synergized with EOs for enhanced bioactivity and stability.	[27,87,118,119]
Electrospinning	Produced nanofibers loaded with EO emulsions for sustained release and antimicrobial activity.	[41,78,113]
Green Synthesis Approaches	Used minimal surfactants for sustainable preparation of EO-based nano emulsions or nanoparticles.	[1,29,45,96]
Pectin-Based Systems	Provided long-term stability and multifunctional applications in food and pharmaceuticals.	[3,6]
Ultrasound–Microwave Combination	Produced emulsions with thermal resistance, improved antibacterial properties, and long shelf life.	[54]
Nanoprecipitation	Efficient for polymer-based EO encapsulation, enabling precise control of release and bioavailability.	[70,91]
GC-MS Analysis and Characterization	Confirmed EO purity and active compounds during preparation; integrated into process optimization.	[18,23,24]
Surfactant-Free Systems	Used essential oils in sustainable emulsions, avoiding synthetic stabilizers while maintaining efficacy.	[45]

**Table 4:** Physicochemical properties of essential oil nano systems.

Property/Characteristic	Description	References
Particle Size	Particle size below 500 nm: ranging from 11–264 nm; smaller sizes correlated with better stability, controlled release, and bioavailability.	[1,5,6,8,11,15,17,24,86,116]
Zeta Potential	High absolute values (−13 to −50 mV) ensured colloidal stability and resistance to aggregation in nano emulsions.	[57,61,91,95]
Encapsulation Efficiency	High efficiencies (70–96%) achieved for improved bioactivity, prolonged release, and reduced degradation of oils.	[33,70]
Thermal Stability	Encapsulation increased thermal stability, protecting oils during processing and storage.	[9,11,111,112,113]
Transparency and Optical Properties	Transparent nano emulsions with low turbidity and minimal light scattering due to uniform, small droplet size.	[3,6,8,117]
Polydispersity Index (PDI)	Narrow size distributions (PDI~0.2–0.3) indicated uniform particle formation and improved stability.	[8,13,20]
Release Kinetics	Controlled release observed with biphasic or sustained profiles, enhancing functional efficacy.	[29,80,120]
Rheological Properties	Nano emulsions exhibited Newtonian or pseudoplastic flow, ensuring stability in various formulations.	[8,20,63,88]
Stability Over Time and Temperature	Stable for months under varying temperatures (1–45 °C) and humidity conditions, with no significant degradation.	[8,42,58,78,121]
Morphology	Uniform spherical particles observed in SEM, TEM, and AFM analyses; improved surface properties confirmed by XRD.	[16,21,45,56,85]
Antioxidant Properties	Antioxidant activity (e.g., DPPH, ABTS scavenging) enhanced by encapsulation, improving food and pharmaceutical applications.	[20,57,83,105]
Antimicrobial/Antifungal Activity	Nano emulsions showed superior antimicrobial/antifungal activity compared to free oils, attributed to better delivery.	[2,49,57,61,71,89,91]
Improved Bioavailability	Enhanced solubility and absorption due to smaller particle sizes and encapsulation, particularly in drug delivery systems.	[17,29,70,73,120]
Food Preservation Properties	Effective in reducing microbial growth, lipid oxidation, and extending shelf life in food systems.	[15,49,59,63,93,102]
Tensile and Barrier Properties	Encapsulation enhanced the mechanical strength, water resistance, and oxygen barrier properties of coatings and films.	[37,88,111,113]
Synergistic Effects in Combinations	Combining essential oils with nanoparticles (e.g., silver and chitosan) boosted antimicrobial efficacy and reduced MIC values.	[16,89,118,119]
Biocompatibility and Safety	Encapsulation minimized toxicity, improved safety profiles, and reduced required dosages for therapeutic applications.	[12,70,71,73,122]

**Table 5:** Proven benefits of taste-masked products.

Unique Benefit	Description	References
Suppression of Bitterness	Taste-masking encapsulation effectively reduced bitterness in bioactive compounds, enhancing overall flavor.	[7,36]
Separation of Active and Flavor Layers	Layered encapsulation techniques isolated taste-masking agents from functional compounds, improving experience.	[37,91]
Prevention of Flavor Degradation	Nanoencapsulation protected flavor profiles from oxidation or reactions with other ingredients during storage.	[8,10]
Enhanced Taste Consistency	Maintained a uniform taste profile across batches by stabilizing volatile components in encapsulated form.	[22]
Suitability for Sensitive Consumers	Targeted for children and older adults, taste-masking formulations made products more palatable and compliant.	[70,120,127]
Dual-Functionality in Food Systems	Combined taste masking with food stabilization, extending shelf life while maintaining acceptable taste.	[3,47,49]
Adaptability to Diverse Formats	Effective across various forms, such as coatings, beverages, and solid foods, without altering organoleptic properties.	[2,15,127]
Healthier Flavor Profiles	Enabled reduction of artificial flavors and sugar while preserving natural taste integrity.	[36]
Preservation of Delicate Aromas	Encapsulation allowed controlled release of pleasant aromas during consumption, elevating sensory experience.	[10,22,34]
Market Expansion for Functional Foods	Facilitated inclusion of strong-tasting bioactive in mainstream food and beverages, widening their market potential.	[2,7,36]
